# Anti-Cancer Activity of an Osthole Derivative, NBM-T-BMX-OS01: Targeting Vascular Endothelial Growth Factor Receptor Signaling and Angiogenesis

**DOI:** 10.1371/journal.pone.0081592

**Published:** 2013-11-27

**Authors:** Hung-Yu Yang, Ya-Fen Hsu, Pei-Ting Chiu, Shiau-Jing Ho, Chi-Han Wang, Chih-Chin Chi, Yu-Han Huang, Cheng-Feng Lee, Ying-Shiuan Li, George Ou, Ming-Jen Hsu

**Affiliations:** 1 Division of Cardiology, Taipei Medical University-Wan Fang Hospital, Taipei, Taiwan; 2 Division of General Surgery, Department of Surgery, Landseed Hospital, Taoyuan, Taiwan; 3 Graduate Institute of Medical Sciences, College of Medicine, Taipei Medical University, Taipei, Taiwan; 4 NatureWise Biotech & Medicals Corporation, Taipei, Taiwan; 5 Department of Pharmacology, School of Medicine, College of Medicine, Taipei Medical University, Taipei, Taiwan; University of Illinois at Chicago, United States of America

## Abstract

Angiogenesis occurs during tissue growth, development and wound healing. It is also required for tumor progression and represents a rational target for therapeutic intervention. NBM-T-BMX-OS01 (BMX), derived from the semisynthesis of osthole, an active ingredient isolated from Chinese herb *Cnidium monnieri* (L.) Cuss., was recently shown to enhance learning and memory in rats. In this study, we characterized the anti-angiogenic activities of NBM-T-BMX-OS01 (BMX) in an effort to develop novel inhibitors to suppress angiogenesis and tumor growth. BMX inhibited vascular endothelial growth factor (VEGF)-induced proliferation, migration and endothelial tube formation in human umbilical endothelial cells (HUVECs). BMX also attenuated VEGF-induced microvessel sprouting from aortic rings *ex vivo* and reduced HCT116 colorectal cancer cells-induced angiogenesis *in vivo*. Moreover, BMX inhibited the phosphorylation of VEGFR2, FAK, Akt and ERK in HUVECs exposed to VEGF. BMX was also shown to inhibit HCT116 cell proliferation and to suppress the growth of subcutaneous xenografts of HCT116 cells *in vivo*. Taken together, this study provides evidence that BMX modulates vascular endothelial cell remodeling and leads to the inhibition of tumor angiogenesis. These results also support the role of BMX as a potential drug candidate and warrant the clinical development in the treatment of cancer.

## Introduction

Angiogenesis is a complex process by which new vessels are formed from preexisting vasculature. It not only contributes to various physiological processes, but also plays an important role in the tumor progression and metastatic spread of tumors [1]–[3]. Tumor vascularity is usually correlated with poor outcome. Tumor-initiated angiogenesis has thus been an attractive target for the development of anti-cancer therapies [4]. During the initial avascular tumor growth, tumor cells outgrow the limitation of diffusion distance to nearby blood vessels and become hypoxic. The balance between angiogenic and anti-angiogenic signaling shifts towards blood vessel formation [5]. This angiogenic switch then activates an array of gene transcriptions and initiates angiogenesis [6]. Numerous angiogenic factors including vascular endothelial growth factor (VEGF) [7], basic fibroblast growth factor (bFGF) [8], [9], epidermal growth factor (EGF) and angiopoietin [10] have been implicated in tumor angiogenesis. Among these angiogenic regulators, VEGF-A, a member of VEGF family, is the most critical and specific mediator that promotes angiogenesis [11], [12]. VEGF-A is required for the chemotaxis and differentiation of endothelial precursor cells, endothelial cell proliferation, vasculogenesis and angiogenic remodeling [13]. Cellular responses to VEGF-A are mediated by the receptor tyrosine kinase VEGFR2 (also known as KDR or Flk-1) on the surface of endothelial cells [14]. Activation of VEGFR2 turns on the signaling cascades including extracellular signal-regulated kinase (ERK), Akt (also known as protein kinase B), focal adhesion kinase (FAK) and Src family kinases [15]. The Akt signaling pathway regulates endothelial cell migration, proliferation and apoptosis [16]. ERK pathway activated by VEGF has been implicated in various cellular activities including proliferation, differentiation, cell motility and survival [17]. FAK also contributes to tumor malignancy [18]. For these reasons, VEGF and VEGFR2 are recognized as attractive targets for therapeutic intervention of cancer [19]. Many strategies to inhibit VEGF signaling are currently being assessed in clinical trials. These include soluble receptors that sequester VEGF [20], antibodies targeting VEGF or VEGFR [21], and small molecule inhibitors of VEGFR2 [22]. Furthermore, some small molecule inhibitors such as sorafenib and sunitinib have already been approved by the United States Food and Drug Administration for treating specific types of cancer [23].

**Figure 1:**
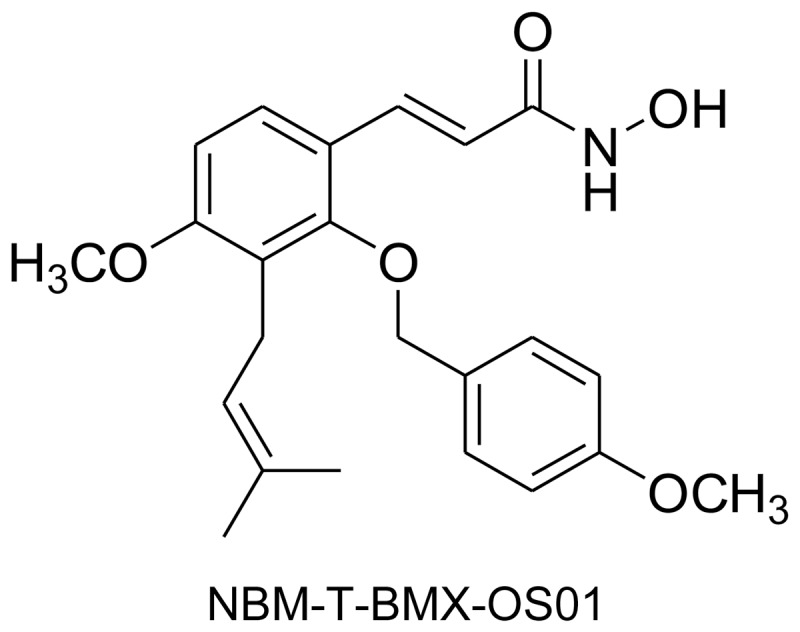
Chemical structure of BMX.

*Cnidium monnieri* (L.) Cuss. has long been widely used in oriental medicine to improve immunity and to alleviate hepatitis. Osthole, a bioactive component extracted from the seeds of *Cnidium monnieri* (L.) Cuss., is thus expected to have immunomodulatory activities. Recent studies also demonstrated that osthole possesses neuroprotective [24], hepatoprotective [25], anti-diabetic [26], and anti-cancer activities [27], [28]. Given osthole's broad spectrum of biological activities, it appears to be a promising lead compound for drug discovery. Recently, NBM-T-BMX-OS01 (BMX) (Fig. 1), a derivative semisynthesized from osthole, was identified as a potent histone deacetylase inhibitor and was shown to enhance learning and memory in rats [29]. In an effort to discover tumor angiogenesis inhibitors, we thus evaluated the anti-angiogenic properties of BMX. In this study, we demonstrated that BMX inhibited VEGF-induced cell proliferation, migration, and tube formation in human umbilical endothelial cells (HUVECs). VEGF-induced phosphorylation of VEGFR2, Src, ERK, Akt and FAK were also suppressed in HUVECs exposed to BMX. By use of HCT116 colorectal cancer cells xenograft angiogenesis model, BMX was further shown to suppress tumor-associated angiogenesis. Furthermore, BMX significantly inhibited HCT116 colorectal cancer cell proliferation and suppressed tumor growth in a xenograft tumor model. Taken together, these results suggest the potential of BMX as a therapeutic agent with dual activity against both tumor proliferation and angiogenesis.

## Materials and Methods

### Reagents

3-[4, 5-dimethylthiazol-2-yl]-2, 5-diphenyltetrazolium bromide (MTT), toluidine blue O, and McCoy5A medium were from Sigma (St Louis, MO). Medium 199 (M199), fetal bovine serum (FBS), and all cell culture reagents were purchased from Invitrogen (Carlsbad, CA). Antibodies against CDK4, VEGFR2, VEGFR2 phosphorylated at tyrosine 1175 (Y1175), VEGFR2 phosphorylated at tyrosine 1214 (Y1214), ERK1/2, ERK1/2 phosphorylated at threonine 202/tyrosine 204 (T202/Y204), Akt, Akt phosphorylated at serine 473 (S473), FAK and FAK phosphorylated at tyrosine 397 (Y397), Src and Src phosphorylated at tyrosine 416 (Y416) were purchased from Cell Signaling (Danvers, MA). Antibodies specific for p21 was purchased from Santa Cruz Biotechnology (Santa Cruz, CA). Antibodies against GAPDH, α-tubulin, survivin and cyclinD1 and anti-mouse and anti-rabbit IgG conjugated peroxidase antibodies were purchased from GeneTex Inc (Irvine, CA). The enhanced chemiluminescence detection kit was from GE Healthcare (Little Chalfont, UK). Cell Proliferation ELISA, BrdU assay kit was acquired from Roche (Indianapolis, IN). All materials for immunoblotting were purchased from GE Healthcare (Little Chalfont, UK). All other chemicals were obtained from Sigma (St. Louis, MO).

### NBM-T-BMX-OS01(BMX)

BMX, (*E*)-2-(4-Methoxybenzyloxy)-3-prenyl-4-methoxy-*N*-hydroxycinamide, was provided by NatureWise Biotech & Medicals Corporation(Taipei, Taiwan), and their purities (>99%) were confirmed by ^1^H-NMR analysis and HPLC. ^1^H NMR spectra were recorded on a Bruker Avance DRX-500 MHz Fourier-transform spectrometer at room temperature. ^1^H NMR (DMSO-*d_6_*, 500 MHz) δ: 7.70 (1H, d, *J* = 16.0 Hz), 7.46 (1H, d, *J* = 8.5 Hz), 7.40 (2H, d, *J* = 8.5 Hz), 6.97 (2H, d, *J* = 8.5 Hz), 6.87 (1H, d, *J* = 8.5 Hz), 6.38 (1H, d, *J* = 16.0 Hz), 5.11–5.05 (1H, m), 4.65 (2H, s), 3.82 (3H, s), 3.78 (3H, s), 3.28 (2H, d, *J* = 7.0 Hz), 1.64 (3H, s), 1.60 (3H, s). Anal. HPLC *t*_R_ = 9.57 min (purity 99.3%). Analytical HPLC UV purity was assessed using a JASCO LC-2000Plus system and the following method. For the method at 210 nm, a Phenomenex Luna 5 µm, 250 mm×4.60 mm C18(2) column was used with a flow rate of 1.0 mL/min and a mobile phase of methanol/water (v/v = 80∶20) over 30 min.

### Ethic statement

This study was carried out in strict accordance with the recommendations in the Guide for the Care and Use of Laboratory Animals of the National Institutes of Health. The protocols as described below were approved by the Taipei Medical University Laboratory Animal Care and Use Committee (Permit Number: LAC-100-0097). All surgery was performed under sodium pentobarbital anesthesia, and all efforts were made to minimize suffering.

### Cell culture

Human umbilical vascular endothelial cells (HUVECs) were obtained from the Bioresource Collection and Research Center (Hsinchu, Taiwan). Cells were maintained in M199 medium containing vascular endothelial cell growth supplement (ECGS) (Millipore), 10% FBS, 5 U/ml heparin, 20 mM HEPES, 100 U/ml of penicillin G, and 100 µg/ml streptomycin in a humidified 37°C incubator. HCT116 colorectal cancer cell line was obtained from the Bioresource Collection and Research Center (Hsinchu, Taiwan). The cells were maintained in McCoy5A containing 10% FBS, 100 U/ml of penicillin G, and 100 µg/ml streptomycin in a humidified 37°C incubator.

### Cell viability assay

Cell viability was measured by the colorimetric 3-(4,5-dimethylthiazol-2-yl)-2,5-diphenyl tetrazolium bromide (MTT) assay as described previously [30].

### Lactate dehydrogenase (LDH) release assay

LDH leakage was measured to quantify cytotoxicity with a CytoTox96 non-radioactive cytotoxicity assay kit (Promega). The supernatants in culture were assayed based on the manufacturer's guidelines. Absorbance was measured at 490 nm in a microplate reader. Lysis buffer added for 10 min was used as a positive control for complete cell necrosis. Data were then calculated as the percentage of lysis buffer-treated group.

### Cell proliferation assay (BrdU incorporation assay)

HUVECs (2×10^4^ per well) were seeded in 96-well tissue culture plates and incubated for 24 h. Cells were then starved in M199 medium containing 2% FBS in the absence of endothelial cell growth supplements for another 16 h. After starvation, cells were pre-treated for 30 min with various concentrations of BMX, followed by the stimulation with VEGF (20 ng/ml) for another 24 h. Cell proliferation was then determined using a Cell Proliferation ELISA, BrdU (colorimetric) kit (Roche) based on the colorimetric detection of the incorporation of BrdU, following the manufacturer instructions.

### Flow cytometric analysis

HUVECs were starved with 2% FBS M199 in the absence of endothelial cell growth supplements for 16 h. The cells were then incubated with BMX at indicated concentrations for 30 min followed by the treatment with VEGF (20 ng/ml) for another 24 h. At the end of the experiments, apoptotic cells were detected by propidium iodide (PI) and annexin V-FITC labeling as described previously [31]. The double labeling was performed at 37°C by treating cells with PI (50 µg/ml) and annexin V-FITC (2 µg/ml) for 15 min in the dark.

### Wound scratch migration assay

After starvation in M199 medium containing 2% FBS for 16 h, monolayer HUVECs were wounded by scratching with pipette tips and washed with PBS. Cells were then treated with various concentrations of BMX in the absence or presence of VEGF (20 ng/mL) for another 16 h. Cells were photographed using phase-contrast microscope with digital camera at 0 h and 16 h after VEGF treatment. The rate of cell migration was determined by counting the number of migrated cells under an inverted contrast phase microscope (Nikon, Japan).

### Transwell invasion assay

Migration assay was done as described previously [32]. Briefly, the bottom face of the filter in the transwell plate (Corning, NY, USA) was coated with 0.2% gelatin. The bottom chambers were filled with M199 medium containing 2% FBS in the presence of VEGF (20 ng/ml) and HUVECs (10^4^ cells per well) in 200 µL M199 medium (without growth factors) were seeded in the top chambers. The top chamber contained vehicle or various concentrations of BMX. Cells were allowed to migrate for 16 h. Non-migrated cells (on the top side of filter) were scraped with a cotton swab, and migrated cells were fixed and stained with 0.5% toluidine blue in 4% paraformaldehyde. The cells were photographed and quantified by counting the number of stained cells under an inverted contrast phase microscope (Nikon, Japan).

### Matrigel tube formation assay

The tube formation assay was performed as described previously [32]. Matrigel, a basement membrane matrix (Becton Dickinson, Mountain View, CA), was polymerized at 37°C for 30 min. HUVECs suspended in M199 medium containing 2% FBS in the presence or absence of VEGF (20 ng/ml) were seeded onto the Matrigel. They were then treated with vehicle or BMX at indicated concentrations. After 16 h, cells were photographed using phase-contrast microscopy.

### Immunoblot analysis

Immunoblot analyses were performed as described previously [30]. Briefly, cells were lysed in an extraction buffer containing 10 mM Tris (pH 7.0), 140 mM NaCl, 2 mM PMSF, 5 mM DTT, 0.5% NP-40, 0.05 mM pepstatin A, and 0.2 mM leupeptin. Samples of equal amounts of protein were subjected to SDS-PAGE and transferred onto a NC membrane which was then incubated in a TBST buffer containing 5% non-fat milk. Proteins were visualized by incubating with specific primary antibodies followed by horseradish peroxidase-conjugated secondary antibodies. Immunoreactivity was detected based on enhanced chemiluminescence per the instructions of the manufacturer. Quantitative data were obtained using a computing densitometer with a scientific imaging system (Biospectrum AC System, UVP).

### Aortic ring sprouting assay

Assay was performed as described previously [32]. Aortic arch was dissected from 8 to 10-week-old Sprague–Dawley rats. After removing the surrounding fibro-adipose tissues and thoroughly rinsing with M199 culture medium, the aortas were cut into 1 mm ring segments. The aortic rings were immersed in Matrigel in the wells of 48-well plate. VEGF (20 ng/ml) with or without BMX was then added to the wells. The aortic rings were cultured in 37°C with 5% CO_2_ and the cultured medium was changed every 3 days. Growing sprouts of endothelial cells were observed and photographed on day 8. The images were photographed under microscope, and sprouting area was determined on the computer-digitized images with Image-Pro Plus (Media Cybernetics) software. The analysis of sprouting area was done by an observer who was blinded to the treatment group. All protocols were approved by the Taipei Medical University Laboratory Animal Care and Use Committee.

### Tumor cell-induced angiogenesis Matrigel plug assay

3-5 week old nude_nu/nu_ mice were obtained from BioLasco (Taipei, Taiwan) and housed in clean specific pathogen free (SPF) rooms. HCT116 cells were harvested and resuspended in PBS. Cells (5×10^6^ cells) in a volume of 150 µl in the presence of heparin (20 U) were mixed with Matrigel (150 µl) and then injected subcutaneously into the right flank of each mouse. After implantation, animals were randomized into the control group and the treatment groups, which received BMX 20 mg/kg/day. The treatment was administered intraperitoneally once daily for 10 days. At the end of treatment, animals were sacrificed, Matrigel plugs were removed and the surrounding tissues trimmed. Hemoglobin levels of the Matrigel plugs were evaluated with Drabkin's reagent kit (Sigma-Aldrich) according to the manufacturer's instructions. The concentration of hemoglobin was calculated based on a set of hemoglobin standards. In addition, paraffin-embedded sections were stained with a CD31-specific antibody (Santa Cruz) to detected the vessel density in Matrigel plug as described previously [33]. All protocols were approved by the Taipei Medical University Laboratory Animal Care and Use Committee.

### Mouse xenograft model

3-5 week old nude_nu/nu_ mice were obtained from BioLasco (Taipei, Taiwan) and housed in clean specific pathogen free (SPF) rooms. HCT116 cells were harvested and resuspended in PBS, and 5×10^6^ cells in a volume of 0.3 mL were injected subcutaneously into the right flank of each mouse. Once the tumor reached approximately 150 mm^3^, animals were randomized into the control group and the treatment group receiving BMX 20 mg/kg/day. The treatment was administered intraperitoneally once daily for 22 days. Tumors were measured every day by a digital caliper. Tumor volume was calculated using the formula *V* (mm^3^) = [*ab* ^2^]×0.52, where *a* is the length and *b* is the width of the tumor [34]. At the end of treatment, animals were sacrificed and tumors were removed and weighed. All protocols were approved by the Taipei Medical University Laboratory Animal Care and Use Committee.

### Statistical analysis

Results are presented as the mean ± S.E. from at least three independent experiments. One-way analysis of variance (ANOVA) was followed by the Newman-Keuls test, when appropriate, to determine the statistical significance of the difference between means. A *p* value of <0.05 was considered statistically significant.

## Results

### BMX inhibits VEGF-induced cell proliferation in HUVECs

**Figure 2:**
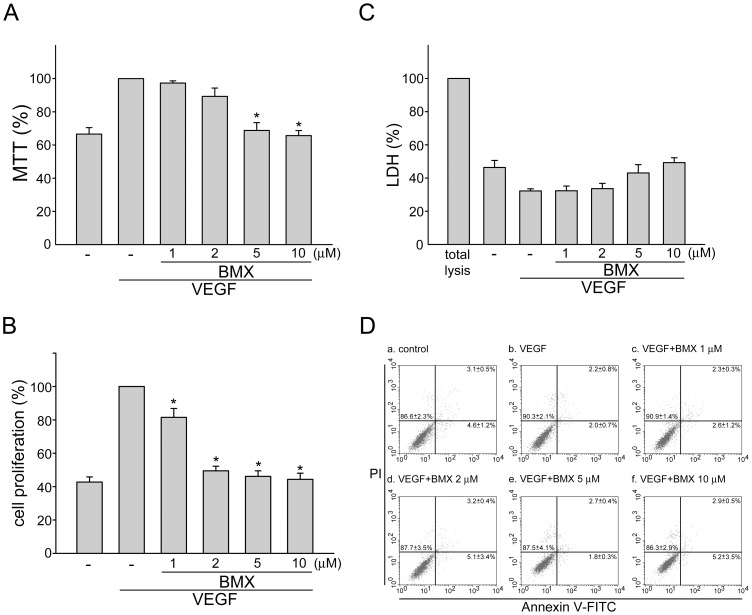
BMX inhibited VEGF-induced cell proliferation in HUVECs. (A) HUVECs were starved in 2% FBS containing medium without ECGS for 16 h. After starvation, cells were pretreated with indicated concentrations of BMX followed by the stimulation with VEGF (20 ng/ml) for another 24 h. Cell viability was then determined by MTT assay. Each column represents the mean ± S.E.M. of five independent experiments performed in duplicate *p<0.05, compared with the group treated with VEGF alone. (B) HUVECs were treated as in (A), and cell proliferation was determined as described in the “*Materials and Methods*” section. Each column represents the mean ± SEM of five independent experiments performed in duplicate. *p<0.05, compared with the group treated with VEGF alone. (C) HUVECs were treated as in (A), and cytotoxicity of BMX was determined by LDH assay. Cells were also treated with cell lysis buffer (total lysis) to serve as positive control. (D) HUVECs were treated as in (A), the percentage of apoptotic cells was then analyzed by flow-cytometric analysis as described in the “*Materials and Methods*” section. The lower left quadrant of each panel (annexin V^−^PI^−^) shows the viable cells, which exclude PI and are negative for annexin V binding. The lower right quadrant (annexin V^+^PI^−^) represents the early apoptotic cells, annexin V positive and PI negative, demonstrating cytoplasmic membrane integrity. The upper right quadrant (annexin V^+^PI^+^) contains advanced apoptotic cells and necrotic cells, which are positive for annexin V binding and for PI uptake. Results shown are representative of four independent experiments.

Endothelial cell proliferation is an essential step in the progress of angiogenesis. To assess the anti-angiogenic activity of BMX (Fig. 1), we first evaluated its inhibitory effects on cell proliferation in VEGF-stimulated HUVECs. Cells were starved with 2% FBS containing medium for 16 h and then stimulated by VEGF (20 ng/ml) in the presence or absence of BMX for another 24 h. As shown in Fig. 2A, treatment of cells with BMX concentration-dependently decreased cell viability in VEGF-stimulated HUVECs as determined by MTT assay. BrdU labeling analysis was then employed to confirm whether BMX-induced decrement in cell viability was attributable to the inhibition of cell proliferation. Fig. 2B shows that the percentage of BrdU-labeled cells significantly increased after a 24 h VEGF treatment when compared to the vehicle treated group. BMX significantly inhibited VEGF-induced cell proliferation in a concentration-dependent manner. To determine whether BMX exerted any cytotoxic effect in HUVECs exposed to VEGF, a LDH assay was employed. As shown in Fig. 2C, treatment of cells with BMX at 10 µM for 24 h did not significantly increase LDH release. We also used flow-cytometric analysis with propidium iodide (PI) and annexin V-FITC labeling to detect apoptosis in VEGF–stimulated cells in the presence of BMX. As shown in Fig. 2D, BMX did not significantly alter the percentage of the annexin V^+^PI^−^ cells (lower right quadrant, early apoptotic cells) and the annexin V^+^PI^+^ cells (upper right quadrant, advanced apoptotic cells and/or necrotic cells). These results suggested that BMX may exert anti-proliferative activity without causing cytolytic or apoptotic effect in HUVECs exposed to VEGF.

### BMX inhibits VEGF-induced migration and capillary structure formation of HUVECs

**Figure 3:**
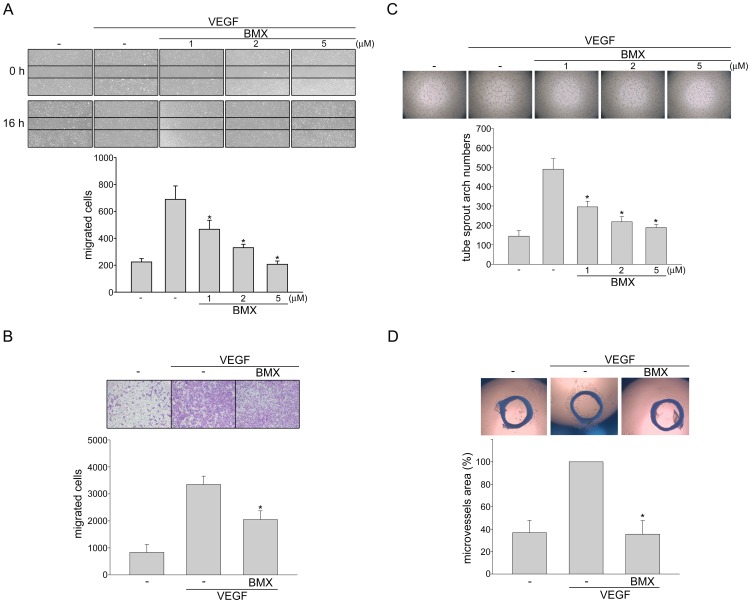
BMX inhibited VEGF-induced migration, tube formation and microvessel sprouting. (A) HUVECs were starved in 2% FBS containing medium without ECGS for 16 h. Cell monolayer were then scratched and treated with vehicle or indicated concentrations of BMX in the presence of VEGF for another 16 h. The number of migrated cells was then determined. Each column represents the mean ± S.E.M. of four independent experiments. *p<0.05, compared with the group treated with VEGF alone. (B) After starvation as described in (A), Cells were then seeded in the top chamber in the absence or presence of BMX (5 µM) using VEGF as chemo-attractant. After 16 h, invaded cells through the gelatin-coated membrane were stained and quantified. Each column represents the mean ± S.E.M. of four independent experiments. *p<0.05, compared with the group treated with VEGF alone. (C) HUVECs were seeded on Matrigel in the presence of VEGF (20 ng/ml) with or without BMX at indicated concentrations. Cells were then photographed under phase-contrast microscopy after 16 h. Bar graphs show compiled data of average sprout arch numbers (n = 4). *p<0.05, compared with the group treated with VEGF alone. (D) Rat aortic rings were placed in Matrigel and treated with VEGF (20 ng/ml) in the presence or absence of BMX (5 µM). The effect of BMX on formation of vessel sprout from various aorta samples was examined on day 8. Bar graphs show compiled data of average microvessels area (n = 4). *p<0.05, compared with the group treated with VEGF alone.

Endothelial cell migration is a pivotal step for angiogenesis [35]. The effect of BMX on HUVEC motility was determined by wound scratch migration and transwell invasion assays. As shown in Fig. 3A, BMX significantly inhibited VEGF-induced HUVEC migration in a concentration-dependent manner. BMX at 5 µM also significantly reduced the number of migrated cells through the transwell membrane barrier when using VEGF as the chemoattractant (Fig. 3B). Angiogenesis is a complex process and tubular formation of endothelial cells is also a key step in angiogenesis. To assess the effect of BMX on the tubular formation of endothelial cells, HUVECs seeded on the surface of Matrigel in the presence of VEGF were treated with either BMX or vehicle as control. Cells in the vehicle-treated group became elongated and formed capillary-like structure within 16 h. VEGF significantly increased capillary-like network. However, treatment of BMX concentration-dependently suppressed the formation of capillary-like network (Fig. 3C). We next explored the potential effects of BMX on VEGF-induced angiogenesis using a rat aortic ring microvessel sprouting assay. As shown in Fig. 3D, VEGF significantly triggered microvessel sprouting, leading to the formation of a complex network of microvessels around the aortic rings, whereas treatment with BMX (5 µM) antagonized the sprouting. These results indicate that BMX may exhibit anti-angiogenic activity through inhibition of cell proliferation, migration and tube formation of endothelial cells.

### BMX inhibits VEGF-induced ERK and Akt phosphorylation

**Figure 4:**
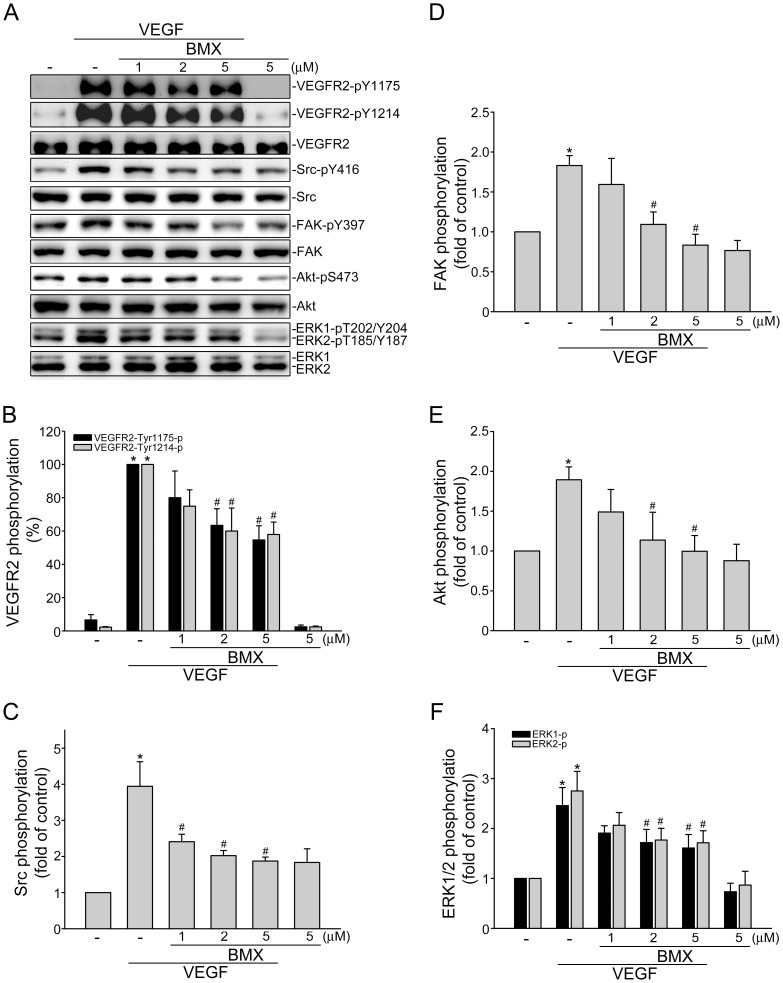
BMX inhibited VEGFR2 signaling pathways in HUVECs. Cells were pretreated with indicated concentrations of BMX for 30(20 ng/ml) for another 5 (VEGFR2 and Src) or 30 (FAK, Akt and ERK1/2) min. Phosphorylation status of VEGFR2, Src, FAK, Akt, and ERK1/2 were then determined by immunoblotting. Figures shown in (A) are representative of four independent experiments with similar results. The compiled results of VEGFR2 Y1175 and Y1214 (B), Src Y416 (C), FAK Y397 (D), Akt S473 (E), and ERK1/2 T202/Y204 (F) phosphorylations are shown. Each column represents the mean ± S.E.M. of four independent experiments. *p<0.05, compared with the control group; #*p*<0.05, compared with the group treated with VEGF alone.

VEGF signaling via VEGFR2 is the most important pathway in inducing endothelial cell proliferation, migration and tube formation, leading to angiogenesis [36]. VEGF binding to VEGFR2 induces VEGFR2 dimerization and subsequently autophosphorylation on its tyrosine residues. Takahashi et al revealed that tyrosine residues 1175 and 1214 are the two major VEGF-dependent autophosphorylation sites of VEGFR2 [37]. We thus determined whether BMX affects VEGFR2 Y1175 and Y 1214 phosphorylation in VEGF-stimulated HUVECs. We also examined the phosphorylation status of Src, Akt, ERK and FAK, which are the essential protein kinases involved in VEGFR2 signaling. As shown in Fig. 4, BMX inhibited VEGFR2 Y1175 and Y1214 phosphorylations in VEGF-stimulated HUVECs (Fig. 4B). BMX was also shown to suppress the phosphorylation of Src (Fig. 4C), FAK (Fig. 4D), Akt (Fig. 4E) and ERK (Fig. 4F) in VEGF-stimulated HUVECs. Together, these results show that BMX exerted its angiogenesis activity by inhibiting VEGFR2 activation and blocking VEGFR2-mediated signaling cascades.

### BMX increases p21^cip/Waf1^ expression, but decreases cyclinD1, CDK4 and survivin expressions

**Figure 5:**
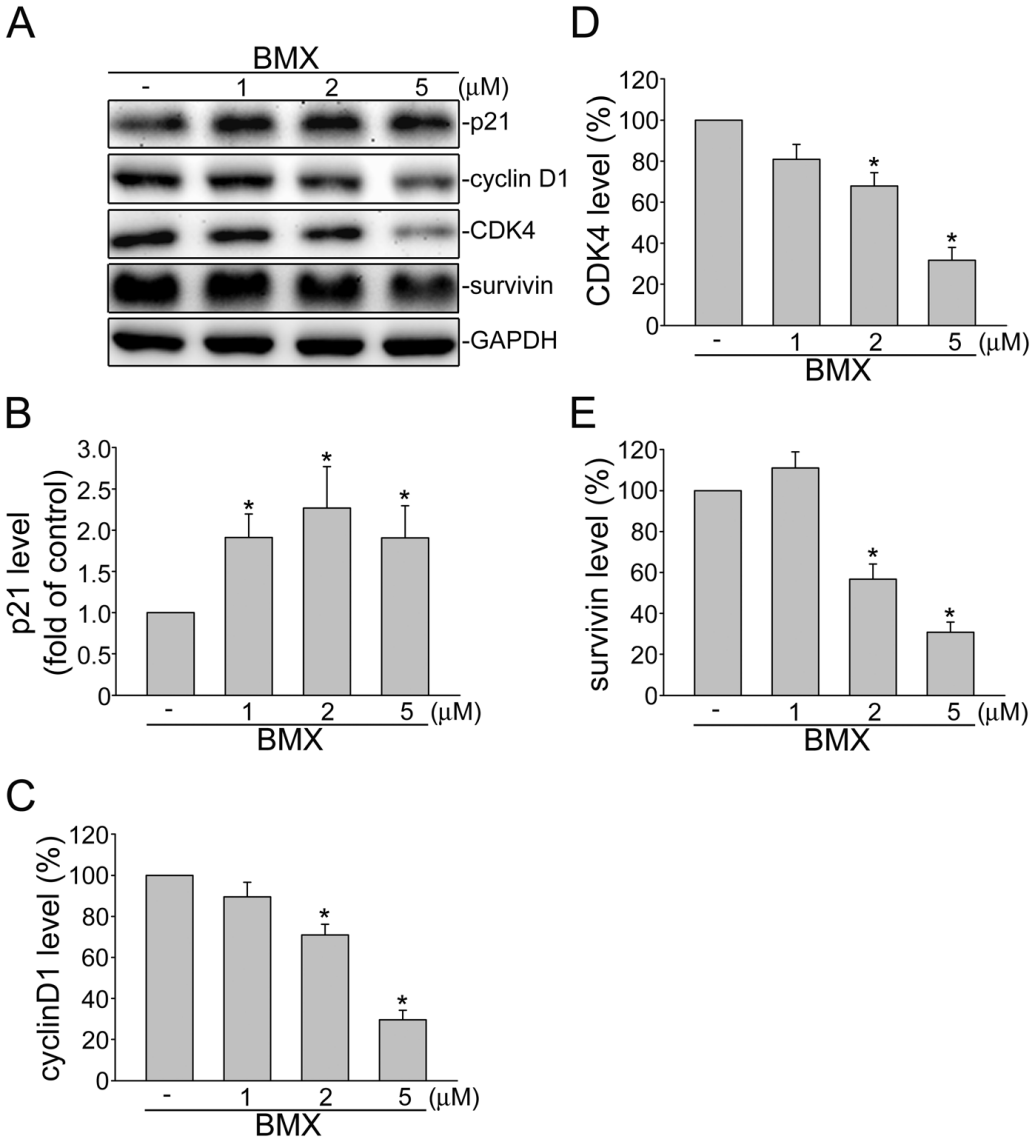
BMX affected the protein levels of p21^cip/Waf1^, cyclinD1, CDK4 and survivin in HUVECs. Cells were treated with indicated concentrations of BMX for 24^cip/Waf1^, cyclinD1, CDK4 and survivin were then determined by immunoblotting. Figures shown in (A) are representative of four independent experiments with similar results. The compiled results of p21^cip/Waf1^ (B), cyclinD1 (C), CDK4 (D), and survivin (E) levels are shown. Each column represents the mean ± S.E.M. of four independent experiments. *p<0.05, compared with the control group

The passage through the cell cycle is regulated by cyclin-CDK complexes, heterodimeric protein kinases [1]–[3]. Many studies have demonstrated that induction or ectopic expression of p21^cip/Waf1^, a negative regulator of CDKs, inhibits cell proliferation and causes differentiation in several cell types. p21^cip/Waf1^ is thus an important regulator of proliferation during development, differentiation, and tumorigenesis [38]. In addition, survivin, an inhibitor of apoptosis (IAP) family member, was found to be over-expressed in human cancers and was reported to play a crucial role in regulating cell cycle progression, apoptosis, and tumorigenesis [39]–[42]. Since BMX suppressed HUVEC proliferation without causing apoptosis as described above, we examined whether BMX affects the protein levels of p21^cip/Waf1^ and survivin in HUVECs. The levels of cell cycle regulatory proteins such as cyclinD1 and CDK4 were also determined in BMX-stimulated HUVECs. As shown in Fig. 5, treatment of HUVECs with BMX for 24 h caused an increase in the protein level of p21^cip/Waf1^ (Fig. 5B). In contrast, the protein levels of cyclin D1 (Fig. 5C), CDK4 (Fig. 5D) and survivin (Fig. 5E) were decreased in BMX-stimulated HUVECs. These results suggest that BMX-inhibited cell proliferation in HUVECs may also be mediated through alterations of the protein levels of p21^cip/Waf1^, cyclinD1, CDK4 and survivin.

### BMX suppressed tumor-induced angiogenesis in nude mice

**Figure 6:**
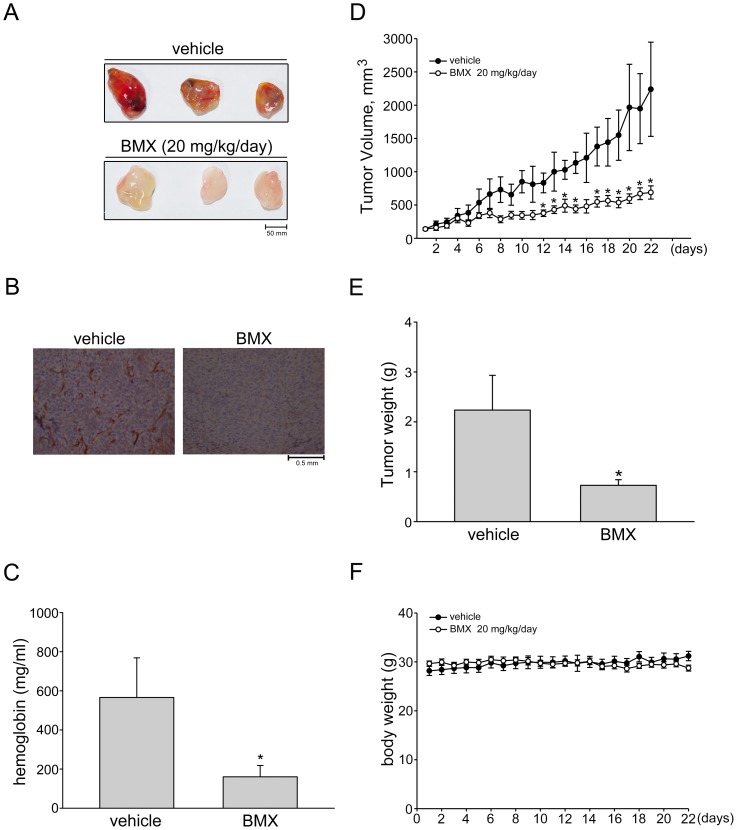
BMX inhibited tumor-induced neovascularization and suppressed *in vivo* tumor growth in nude mice. (A) HCT 116 colorectal cancer cells were mixed with Matrigel and then injected subcutaneously into the right flank of nude mice. After implantation, animals were treated intraperitoneally with vehicle or BMX 20 mg/kg/day for 10 days. Matrigel plugs removed from the mice administered intraperitoneally with vehicle or BMX were shown. (B) The blood vessels in the Matrigel plug were stained with anti-CD31 antibody as described in the “*Materials and Methods*” section. Images of immunohistochemical staining representative of three independent Matrigel plug with similar results are shown. (C) Hemoglobin levels in the Matrigel plug shown in (A) were quantified. Each column represents the mean ± S.E.M. of six plugs in each group (^*^p<0.05 as compared with the vehicle-treated control group, n = 5). (C) Nude mice bearing xenografts of HCT116 colorectal cancer cells were treated intraperitoneally with BMX 20 mg/kg/day for 22 days. The control group received vehicle only. Tumor volumes were calculated as described in the *Materials and Methods* section. Values represents the mean ± S.E.M. (^*^p<0.05 as compared with the vehicle-treated control group, n = 5). (D) After 22 days of treatment as in (C), mice were sacrificed and tumors were dissected and weighted. Each column represents the mean ± S.E.M. (^*^p<0.05 as compared with the vehicle-treated control group, n = 5). (E) The body weights of the nude mice were examined within 22 days treatment of vehicle or BMX. Values represent the mean ± S.E.M.

Next, we used a tumor-induced angiogenesis model to further investigate whether BMX inhibits tumor-induced angiogenesis. HCT116 colorectal cancer cells were mixed with Matrigel and injected into the flanks of mice. Gel plugs were harvested 10 days after implantation. As shown in Fig. 6A, HCT116 cells profoundly induced blood vessel formation in the plug (Fig. 6A). However, the pale color of the plugs removed from the mice administered intraperitoneally with BMX (20 mg/kg/day) indicated that HCT116 cells induced less neovascularization over the 10-day period. We performed CD31 immunohistochemical staining to detect the microvessel density in plugs. As shown in Fig. 6B, plugs derived from BMX-treated mice exhibited a decrease in microvessel density when compared with those from vehicle-treated control mice. We also quantified the level of angiogenesis by determining the hemoglobin content of the plugs. A marked reduction in neovascularization was shown in plugs from BMX-treated mice when compared with those from vehicle-treated control mice (Fig. 6C). These data indicate that systemic administration with BMX significantly suppressed angiogenesis in this *in vivo* assay.

### BMX suppressed tumor growth in a mouse xenograft model

Angiogenesis is the key step in tumor growth, which provides necessary oxygen and nutrients for the tumor [43]. We next investigated the effect of BMX on tumor growth using a mouse xenograft colorectal tumor model. HCT116 colorectal cancer cells were injected into the flanks of mice. After allowing the tumors to grow subcutaneously to an average size of about 150 mm^3^, animals were treated with either vehicle or BMX (20 mg/kg/day) by daily intraperitoneal injections (I.P.) for 22 days. At the end of 22 days, mice were sacrificed and tissue samples were collected. BMX-treated mice had smaller tumor volume (Fig. 6D) and tumor weight (Fig. 6E) comparing to controls. These results suggest that BMX treatment is capable of attenuating the tumor growth in the mouse xenograft colorectal tumor model. In addition, injection of BMX at 20 mg/kg/day had no significant effect on mouse body weight as compared to the vehicle-treated control group (Fig. 6F). This might imply that BMX possesses little side effects at the therapeutic dose.

### BMX inhibited cell proliferation in HCT116 colorectal cancer cells

**Figure 7:**
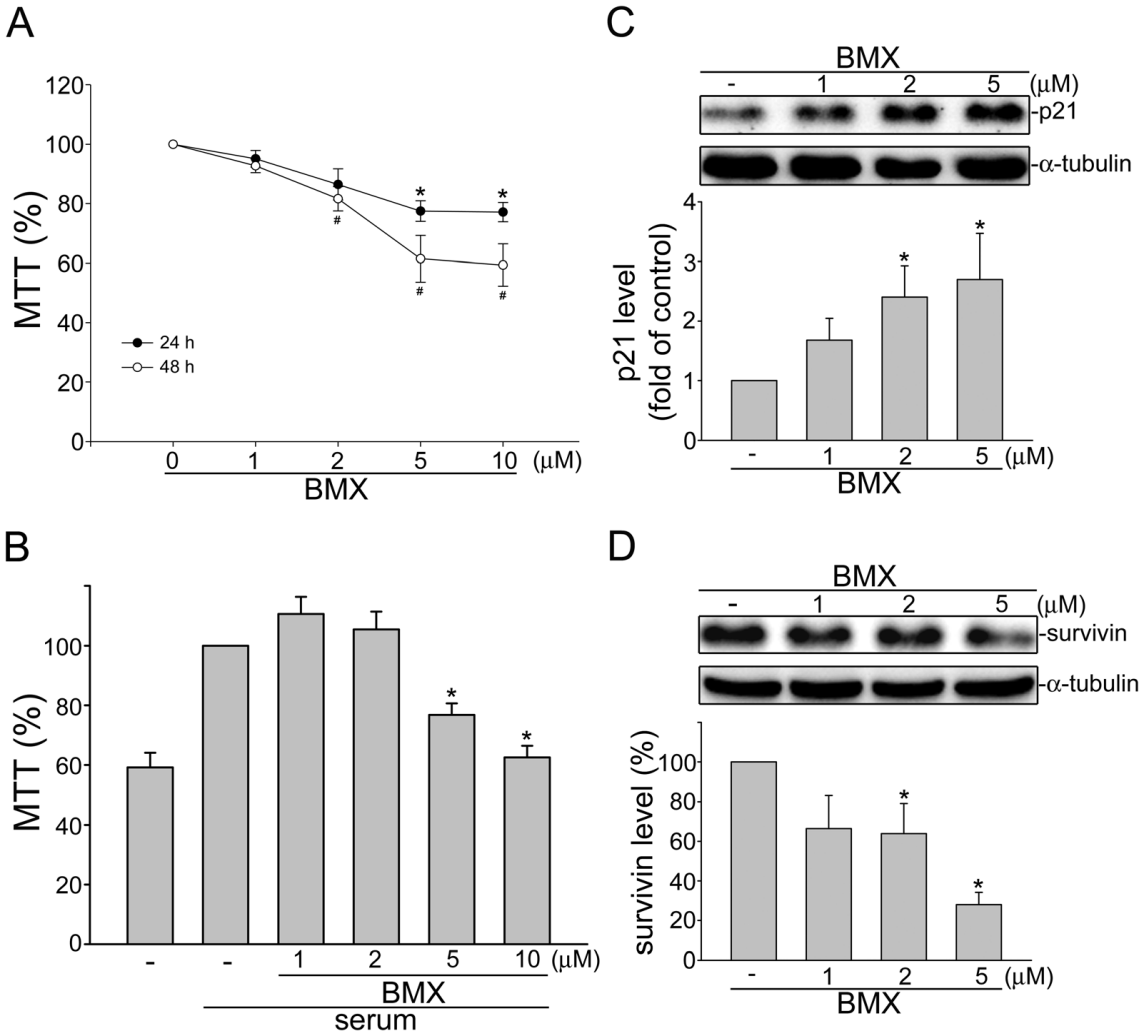
BMX inhibited serum-induced cell proliferation in HCT116 cells. (A) HCT116 cells were treated with vehicle or BMX at indicated concentrations for 24 or 28 h. Cell viability was then determined by MTT assay. Each column represents the mean ± S.E.M. of seven independent experiments performed in duplicate. (^*^p<0.05 as compared with the vehicle-treated control group) (B) After starvation in serum free medium for 24 h, HCT116 cells were treated with indicated concentrations of BMX in the presence of serum (10% FBS) for another 24 h. Cell viability was then determined by MTT assay. Each column represents the mean ± S.E.M. of five independent experiments performed in duplicate. (^*^p<0.05 as compared with the vehicle-treated control group in the presence of serum) Cells were treated for 24 h with vehicle or BMX at indicated concentrations. Protein levels of p21 (C) and survivin (D) were then determined by immunoblotting. Compiled results are shown at the bottom of the chart. Each column represents the mean ± S.E.M. of five independent experiments. (^*^p<0.05 as compared with the vehicle-treated control group).

In addition to angiogenesis, tumor cells proliferation is also required for tumor progression, we thus used MTT assay to examine cell viability and proliferation in HCT116 cells exposed to BMX. As shown in Fig. 7A, BMX decreased cell viability in time- and concentration-dependent manners. To determine if the action of BMX in decreasing HCT116 viability was attributable to the inhibition of cell proliferation, cells were starved with serum-free medium for 24 h, and then incubated in serum (10% FBS) containing medium in the absence or presence of BMX for another 24 h. As shown in Fig. 7B, treatment of cells with BMX significantly decreased serum-induced cell proliferation of HCT116 colorectal cancer cells. We also examined the protein levels of p21^cip/Waf1^ and survivin in HCT116 cells in the presence of BMX. As shown in Fig. 7C and 7D, BMX significantly increased p21^cip/Waf1^ level (Fig. 7C), but suppressed survivin level (Fig. 7D).

## Discussion

Angiogenesis plays a critical role in tumor growth and metastasis [44], [45]. The inhibition of tumor angiogenesis has therefore become an important therapeutic strategy for cancer treatment. Several inhibitors of tumor angiogenesis have been shown successfully attenuated tumor growth and metastasis in the treatment of solid tumors [1], [46]. These observations led to increased efforts to discover novel angiogenesis inhibitors. There has been growing evidence of the beneficial effects of bioactive components from dietary sources or traditional Chinese medicine for the treatment of cancer [47]. Therefore, these bioactive ingredients might be suitable lead compound candidates for anti-tumor drug development. BMX, an osthole derivative, which was developed by structurally modifying osthole, was recently identified as a potent histone deacetylase inhibitor and was shown to enhance learning and memory in rats [29]. However, osthole, a bioactive compound isolated from medical plants, has been demonstrated to possess anti-tumor activity [27]. BMX is thus suggested to exhibit anti-tumor property in addition to the effects on learning and memory. In this study, we investigated and characterized anti-angiogenic and anti-tumor properties of BMX. We demonstrated that BMX inhibits VEGF-induced cell proliferation, migration, and tube formation of HUVECs. BMX was also shown to suppress tumor-induced angiogenesis and tumor growth *in vivo*. Furthermore, we demonstrated for the first time that suppression of VEGFR2-mediated signaling such as Src, Akt, ERK and FAK may contribute to BMX-induced inhibition on angiogenesis. These results indicate that BMX is an angiogenesis inhibitor.

VEGFR2 plays a pivotal role in mediating angiogenic effects of VEGF in endothelial cells. There are several VEGFR2 residues that become phosphorylated upon VEGF exposure. Among these residues, VEGFR2 Y1175 is the most important in angiogenesis [48]. VEGFR2-mediated activation of Src, Akt and ERK was shown to contribute to VEGF-induced endothelial cell migration, proliferation and survival [16], [17], [48]–[50]. VEGF phosphorylation of VEGFR2 also leads to the activation of FAK, which participates in focal adhesion and regulates cell motility [22]. Suppressing VEGFR2 signaling is thus thought to be a promising strategy for cancer therapy [32], [51]. In agreement with these observations, we noted in this study that BMX attenuated not only Y1175, but also Y1214 phosphorylation of VEGFR2 in HUVECs exposed to VEGF. BMX also inhibited VEGF-induced Src, Akt, ERK and FAK phosphorylation. Results demonstrated by *in vivo* Matrigel plug assay also suggest that BMX attenuates HCT116 colorectal cancer cells-induced angiogenesis. Taken together, we believe that the inhibitory effects of BMX on VEGFR2 phosphorylation and the subsequent signaling cascades may be responsible for its anti-angiogenic effects. The molecular mechanism underlying BMX-induced dephosphorylation of VEGFR2 has not been delineated but is likely to involve BMX activation of protein tyrosine phosphatase (PTP). Protein phosphatases constitute the group of enzymes that positively and negatively regulate growth factor effects. There are many PTPs including receptor type and non-receptor type, which are expressed in endothelial cells [52]. Among these PTPs, density enhanced phosphatase (DEP)-1 and VE-PTP have been reported to play a negative regulatory role in VEGFR2 signaling. DEP-1 [53] or VE-PTP [54] deletion leads to increased VEGFR2 phosphorylation with consequent activation of downstream signaling pathways. PTP1B was shown to negatively regulate VEGF signaling by dephosphorylating VEGFR2 [55]. In addition, VEGFR2 phosphorylation may be also regulated by members of the SH2 domain-containing protein tyrosine phosphatase (SHP) family. Sugano et al [56] demonstrated that knockdown of SHP-1 by siRNA accelerates angiogenesis in a rat model. Further investigations are needed to elucidate whether PTPs such as DEP-1, VE-PTP, SHP-1 or PTP1B contribute to the anti-angiogenic actions of BMX.

In *in vivo* murine xenograft model, we demonstrated the effectiveness of BMX, an osthole derivative, in suppressing tumor progression. Osthole has recently been reported to inhibit HepG2 human hepatocellular carcinoma cellular growth through the induction of cell cycle arrest and apoptosis. This is the result of suppressing NF-κB activity and promoting apoptosis-related genes expressions [27]. Osthole was also shown to induce apoptosis in A549 human lung cancer cells by regulating the expressions of Bcl-2 and Bax [57]. Xu et al [58] further demonstrated that osthole inhibited the migration and invasion of A549 cells by reducing the matrix metalloproteinase-2 (MMP-2) and MMP-9 levels. It is conceivable that a drug may target both the tumor cell and the proliferating endothelial cell. In addition to blocking VEGFR2 signaling and exhibiting anti-angiogenic activity, we noted in this study that BMX also significantly suppressed cell proliferation in HCT116 human colorectal cancer cells. Treatment of HUVECs and HCT116 cells with BMX significantly elicited the cell cycle regulator, p21. The elevated levels of p21 by BMX may provide a barrier to proliferation by blocking cell cycle machinery. Survivin down-regulation may also contribute to the anti-proliferative actions of BMX since survivin plays an important role in mitosis [59]. However, only subtle BMX-induced cell apoptosis was observed in HCT116 cells, even at high concentration (10 µM) (data not shown). It suggests that the anti-proliferative and apoptotic effects of osthole and its derivative, BMX, on tumor cells may vary among different types of tumor. Further investigations may be needed to clarify whether BMX inhibits cell proliferation or induces apoptosis in other types of tumors. In addition, BMX was shown to reduce cyclinD1 and CDK4 levels in HUVECs. This is likely the mechanism by which BMX suppressed cell proliferation. It is also worthwhile to explore whether alteration of other cell cycle regulatory proteins contributes to anti-proliferative actions of BMX and whether BMX affects tumor invasion and metastasis.

In summary, we have demonstrated that BMX exhibits dual functions, including suppression of angiogenesis by targeting VEGFR2 signaling and inhibition of tumor cell proliferation. These results support the notion that BMX may be a good candidate of anti-angiogenic agent and a valuable lead compound in the development of anticancer therapy.
